# On the Role of a Conserved Methionine in the Na^+^-Coupling Mechanism of a Neurotransmitter Transporter Homolog

**DOI:** 10.1007/s11064-021-03253-w

**Published:** 2021-02-09

**Authors:** Wenchang Zhou, Gianluca Trinco, Dirk J. Slotboom, Lucy R. Forrest, José D. Faraldo-Gómez

**Affiliations:** 1 Theoretical Molecular Biophysics Laboratory, National Heart, Lung and Blood Institute, National Institutes of Health, Bethesda, MD 20892 USA; 2 Groningen Biomolecular Sciences and Biotechnology Institute, Zernike Institute for Advanced Materials, University of Groningen, Groningen, The Netherlands; 3 Computational Structural Biology Section, National Institute of Neurological Disorders and Stroke, National Institutes of Health, Bethesda, MD 20892 USA

**Keywords:** Transport stoichiometry, Molecular dynamics simulations, Cation-methionine interactions, Sulfur polarization

## Abstract

Excitatory amino acid transporters (EAAT) play a key role in glutamatergic synaptic communication. Driven by transmembrane cation gradients, these transporters catalyze the reuptake of glutamate from the synaptic cleft once this neurotransmitter has been utilized for signaling. Two decades ago, pioneering studies in the Kanner lab identified a conserved methionine within the transmembrane domain as key for substrate turnover rate and specificity; later structural work, particularly for the prokaryotic homologs Glt_Ph_ and Glt_Tk_, revealed that this methionine is involved in the coordination of one of the three Na^+^ ions that are co-transported with the substrate. Albeit extremely atypical, the existence of this interaction is consistent with biophysical analyses of Glt_Ph_ showing that mutations of this methionine diminish the binding cooperativity between substrates and Na^+^. It has been unclear, however, whether this intriguing methionine influences the thermodynamics of the transport reaction, i.e., its substrate:ion stoichiometry, or whether it simply fosters a specific kinetics in the binding reaction, which, while influential for the turnover rate, do not fundamentally explain the ion-coupling mechanism of this class of transporters. Here, studies of Glt_Tk_ using experimental and computational methods independently arrive at the conclusion that the latter hypothesis is the most plausible, and lay the groundwork for future efforts to uncover the underlying mechanism.

**Supplementary Information:**

The online version of this article (10.1007/s11064-021-03253-w) contains supplementary material, which is available to authorized users.

## Introduction

**Fig. 1:**
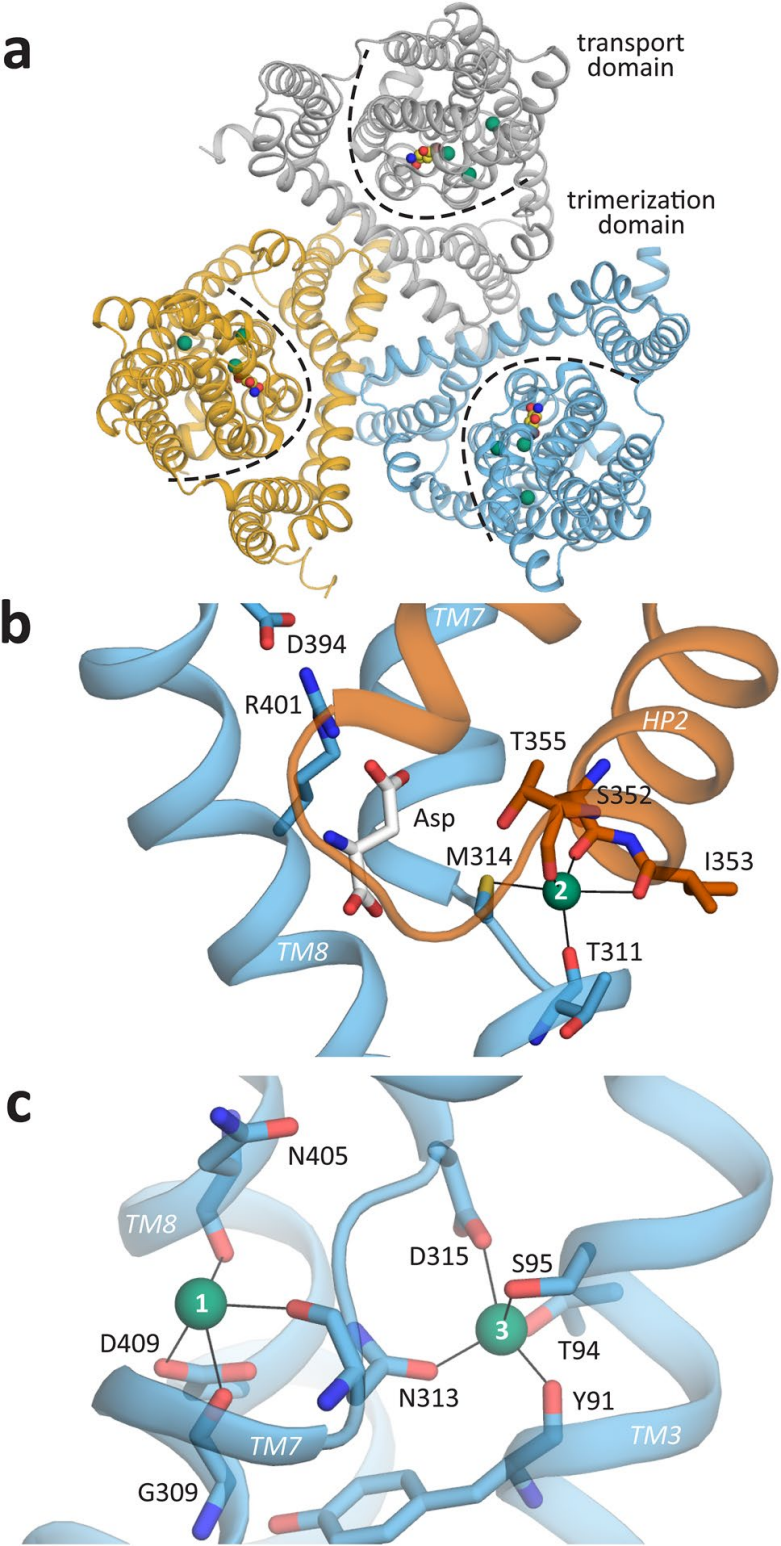
Structure and binding sites of Glt_Tk_. **a** The structure of the holo trimer (PDB entry 5E9S), in an outward-facing conformation, is viewed from the extracellular side and shown as cartoon helices, with each subunit in a different color. Dashed lines indicate the boundaries between the transport domain and trimerization domain in each protomer. Sodium ions (*green*) and L-aspartate (*yellow, blue and red*) are shown as spheres. **b** Close-up view of the binding site for L-aspartate (*sticks with carbon atoms in white*), and of the Na^+^ binding site referred to as Na2 (*green sphere*), with key residues, including Met314, shown as sticks and labelled. Hairpin HP2 (*orange*) occludes access to these sites from the extracellular side. Thin black lines indicate oxygen- or sulfur-ion coordination. **c** Close-up of the Na1 and Na3 sites, deeper into the structure of the transport domain, showing key residues involved in Na^+^ coordination

Glutamatergic synapses are the primary excitatory synapses in the brain and are thought to be essential for learning and memory. In this form of chemical signaling, glutamate is released by the presynaptic nerve terminal, activating receptor proteins on the surface of the post-synaptic neuron. Excessive quantities of glutamate in the synaptic cleft, however, can be cytotoxic and are associated with traumatic events such as stroke (reviewed in [1, 2]). Reuptake of glutamate is thus key to maintaining healthy levels, a task that falls largely to membrane transporters belonging to the SLC1 family, also known as excitatory amino acid transporters (EAAT) in the transporter classification database (TCDB) family 2.A.23 [3, 4]. Structures of prokaryotic SLC1 homologues, including Glt_Ph_ and Glt_Tk_, from *Pyrococcus horikoshii* and *Thermococcus kodakarensis*, respectively [5–7], and of a thermally-stabilized mutant of EAAT1 (SLC1A1 or GLAST) [8], reveal a trimeric assembly, where each protomer contains eight transmembrane (TM) segments and two helical hairpins, HP1 and HP2. The three protomers interact through their so-called trimerization domains (Fig. 1a), whose relative orientation remains constant during transport [9]. By contrast, the substrate-binding transport domains, in the periphery of the complex, (Fig. 1a) undergo dramatic conformational changes, often referred to as elevator-like movements, in order to allow alternating access to the binding sites from each side of the membrane [10–14].

Like their mammalian cousins, Glt_Ph_ and Glt_Tk_ both require three sodium ions to drive transport of the substrate dicarboxylic acid [15–18]. However, they also differ in that they have a higher affinity for L-aspartate than for L-glutamate, and do not require antiport of potassium nor co-transport of a proton [5, 19, 20]. The binding sites for sodium and substrate are buried beneath HP2 in the core of the transport domain, and in close proximity to one another (Fig. 1b, c) but not in direct contact. The Na^+^ binding site furthest from the extracellular surface of the protein, denominated Na3, is formed by Tyr91, Thr94 and Ser95 in TM3 (residue numbering in Glt_Tk_ will be used hereafter) as well as Asn313 and Asp315 from TM7 (Fig. 1c). The latter are part of a conserved sequence motif, NMDGT, which the Kanner lab revealed to be essential for the transport functionality of mammalian transporters [21–24]. Asn313 also contributes a backbone carbonyl atom to the so-called Na1 site, as does Gly309 from TM7, the side chain of Asp409, and the backbone carbonyl of Asn405, both in TM8 (Fig. 1c). The Na2 site is the most proximal to the extracellular space, and also to the substrate binding site. Ion coordination at this site differs from that of Na1 and Na3, in that it does not involve a direct contact with an acidic sidechain, as is typical among Na^+^-coupled transporters [25–29]. Instead, it involves only four backbone carbonyl oxygens from residues in HP2 (Ser352, Ile353 and Thr355) and TM7 (Thr311), and most strikingly, the sidechain of Met314, of the NMDGT motif (Fig. 1b).

This methionine is particularly intriguing. In the known EAAT structures, it separates the Na^+^ ion in the Na2 site from the substrate, when both are bound. However, such direct methionine-Na^+^ contacts are extraordinarily rare: a search of the Protein Data Bank [30] reveals only three other structures that appear to feature this interaction, but none of those proteins are known to be Na^+^ dependent, so these assignments are not conclusive (Table S1). Yet Met314 is conserved in all known sodium-coupled transporters in the SLC1 family; interestingly, though, it is substituted by Leu in proton-coupled transporters such as *E. coli* GltP [31] and *B. subtilis* DctA [32]. Studies in the Kanner lab have examined the effects resulting from mutation of this conserved methionine in Na^+^-dependent mammalian transporters (Met397 in EAAT2, SLC1A2 or GLT-1, and Met367 in EAAT3, SLC1A3 or EAAC1). Interestingly, substitutions by Cys, Leu, Ala or Ser were observed to severely diminish the rate of substrate uptake and to alter its dependence on Na^+^ concentration [21, 23, 33], in a manner that suggests Na^+^ binding is partially impaired. For example, for Cys, Ser and Leu substitutions, Na^+^-driven uptake of L-[^3^H]-glutamate and D-[^3^H]-aspartate was reduced relative to the wild type transporter by 10–20-fold, reflecting concomitant changes in *K*_m_ measured using steady-state transport currents [33]. Moreover, the K_0.5_ for Na^+^ required to activate those steady-state currents was increased by at least 2.5-fold relative to the wild type transporter [33]. More recent studies of Glt_Ph_ and Glt_Tk_ have dissected the interdependence between substrate and Na^+^ binding in detail, concluding that these processes are highly cooperative. Specifically, existing data indicates that two Na^+^ ions must bind before the substrate can be recognized [34–36], presumably to sites Na1 and Na3 in the outward-facing conformation. Binding of substrate and of the third ion to the Na2 site would follow. However, this cooperativity is greatly suppressed when Met314 (Met311 in Glt_Ph_) is replaced by Ala or Leu [37], as is the substrate affinity. Although no structures have been reported for any of these mutants, Trp fluorescence measurements show that the pattern of conformational changes in HP2 in response to ion and substrate binding [19, 37] is also subtly altered upon mutation of Met314 [38, 39].

To our knowledge, the precise explanation for these intriguing effects on uptake rates and sodium dependence remains unclear. A plausible hypothesis is that Met314 contributes to defining the Na^+^ stoichiometry of the transport reaction; by perturbing the Na2 site, mutation of Met314 would logically slow down the reaction for a given Na^+^ concentration gradient, and also partially decouple substrate and Na^+^ binding, as observed. Alternatively, it is also plausible that the nature of these effects is primarily kinetic: Met314 might confer a specific order to the occupancy of the substrate and Na2 sites, and contribute to defining their respective affinities, but wild-type and mutant transporters would be, from a thermodynamic and mechanistic standpoint, fundamentally the same. Here we examine the role of Met314 for the Glt_Tk_ transporter through biochemical stoichiometry assays, molecular dynamics simulations, and quantum mechanical calculations.

## Materials and Methods

### Analysis of the Protein Data Bank

Examples of methionine or cysteine side chains interacting with a sodium ion in known structures were assessed by searching the Protein Data Bank (PDB) as of 2020-10-14. The search was focused on biological assemblies with resolution 3.0 Å or better in which sodium ions are present. Within that dataset, the distances between the sodium ions and the sulfur atoms of either a Met or Cys side chain were evaluated. A contact was identified when that distance was 3.5 Å or smaller.

### Quantum Mechanics (QM) Calculations and NBFIX Corrections

All QM calculations were carried out with Gaussian09 (Gaussian Inc, Wallingford CT, USA). Second-order Møller-Plesset perturbation theory (MP2) with the AUG-cc-pVTZ basis set was used for both geometry optimizations and potential-energy calculations. As analogs of the methionine and cysteine sidechains, we used methylthioethane and methanethiol, respectively. To evaluate the interaction between each analog and a Na^+^ ion, the potential energy was calculated for a series of structures in which the distance between the sulfur atom and the ion varies from 2 to 7 Å in 0.1 Å increments. To evaluate the nature of any polarization effects, the charge that is effectively localized in each atom of the analog was evaluated with Natural Bond Orbital (NBO) analysis for three of those structures, at 7.0, 4.0 and 2.8 Å, respectively. The associated electrostatic potentials were then calculated using APBS (www.poissonboltzmann.org) and mapped onto the molecular surface of each analog with PyMol v2.4 (Schrödinger, Inc).

To develop the NBFIX corrections for Met and Cys, analogous sets of 51 configurations were evaluated with NAMD2.9 [40] and the CHARMM36 force field [41, 42], obtaining a potential-energy curve for the default set of Lennard–Jones parameters for each analog. The parameters that specifically describe the S-Na^+^ interaction in each case, and *R*_min_, were then varied so as to ideally match the MP2 potential energy curves. The range of values scanned for was –5.0 to –14.0 kcal/mol, and 2.7 to 2.8 Å for *R*_min_.

### Molecular Dynamics (MD) Simulations

All MD simulations were carried out with NAMD2.9 or 2.12 [40] using the CHARMM36 force field [41, 42], supplemented by the NBFIX corrections described above, wherever noted. The simulations were carried out with periodic boundary conditions, constant temperature (298 K), constant semi-isotropic pressure (1 atm), and using an integration time-step of 2 fs. Electrostatic interactions were calculated using the Particle-Mesh Ewald method [43], with a real-space cut-off of 12 Å. Van der Waals interactions were computed with a Lennard–Jones potential, cut off at 12 Å with a smooth switching function taking effect at 10 Å. Analysis was carried out using VMD [44] on snapshots taken every 2 ps.

Simulations of holo- and apo-state Glt_Tk_ in the outward-facing conformation were based on PDB entries 5E9S and 5DWY, respectively [19]. In both cases, the protein was embedded in a pre-equilibrated, hydrated 1-palmitoyl-2-oleoyl-*sn*-glycero-3-phosphocholine (POPC) lipid bilayer using GRIFFIN [45]. Default ionization states were used, and water molecules were added into hydrophilic cavities within the protein using Dowser [46]. The size of the resulting systems is approximately 136 × 136 × 87 Å and each contains ~ 400 POPC molecules and ~ 32,000 water molecules. The simulation systems were equilibrated following a staged protocol comprising a series of restrained simulations. The protocol consists of both positional and conformational restraints, gradually weakened over 150 ns and individually applied to protein side chains and backbone as well as the substrate and Na^+^ ions resolved by X-ray crystallography. A trajectory free of any restraints was calculated subsequently. For both the holo state, either with default or NBFIX-corrected CHARMM36, and the apo state, the trajectories calculated are 2 μs long.

An analogous protocol was followed to simulate two variants of the holo-state structure in which the sidechain of Met314 was replaced by Ala or Cys in the three protomers. These trajectories are 500 ns long.

Two additional simulations, each 1 μs long, were carried out in which Na^+^ ions were sequentially added to the Na3 and Na1 sites in the wild type apo state. Specifically, we extracted the apo-state configuration observed after 1 μs of MD simulation and added Na^+^ ions to the three Na3 sites in the trimer by slowly transforming a nearby water molecule into Na^+^, using the Free Energy Perturbation module in NAMD [40]. In this alchemical transformation, carried out over 50 ns, the appearing sodium ion and the vanishing water molecule were confined to the Na3 site using upper-bound distance restraints. Simultaneously, a Na^+^ ion in the solution was transformed into a water molecule, one for each protomer, so as to preserve the net charge of the simulation system. After the transformation was completed, a trajectory of 1 μs was calculated without any restraints. At the end of this trajectory, Na^+^ ions were then added to the three Na1 sites in the trimer using the same protocol, and subsequently, a 1 μs-long trajectory was calculated.

### Mutagenesis, Expression and Purification

Mutagenesis was carried out using the QuickChange protocol (using primers CAACCATTAATTGCGATGGCACCGCAC and TGCGGTGCCATCGCAATTAATGGTTG for M314C; and CAACCATTAATAGCGATGG CACCGCAC and TGCGGTGCCATCGCTATTAATGGTTG for M314S) on the expression plasmid described in [6]. The constructs were verified by DNA sequencing and transformed in *E. coli* MC1061 cells. Expression and purification were carried out as previously described [47].

### Reconstitution into Proteoliposomes

A solution of *E. coli* total lipid extract (20 mg ml^−1^ in 50 mM KPi, pH 7.0) was extruded with a 400-nm-diameter polycarbonate filter (Avestin, 11 passages) and diluted with the same buffer to a final concentration of 5 mg ml^−1^. The lipid mixture was destabilized with 10% Triton-X100. Purified Glt_Tk_ and the destabilized lipids were mixed in a ratio of 1:250 (protein: lipid) and incubated at room temperature for 30 min. Bio-beads were added four times (25 mg ml^−1^, 15 mg ml^−1^, 19 mg ml^−1^, 29 mg ml^−1^ lipid solution) after 0.5 h, 1 h, overnight and 2 h incubation, respectively, on a rocking platform at 4 °C. The Bio-beads were removed by passage over an empty Poly-Prep column (Bio-Rad). The proteoliposomes were collected by centrifugation (20 min, 298,906 g, 4 °C), subsequently resuspended in 50 mM KPi, pH 7.0 to the lipid concentration of 20 mg ml^−1^ and freeze-thawed for three cycles. The proteoliposomes were stored in liquid nitrogen until subsequent experiments.

### Measuring Transporter Equilibrium Potentials

Stored proteoliposomes were thawed and collected by centrifugation (20 min, 298,906 g, 4 °C), the supernatant was discarded and the proteoliposomes were resuspended to a concentration of 10 mg ml^−1^ of lipids in buffer containing 20 mM Hepes/Tris, pH 7.5, 200 mM NaCl, 50 mM KCl, 10 μM L-aspartate (containing 1 μM [^3^H]-L-aspartate). The internal buffer was exchanged by three cycles of freezing in liquid nitrogen and thawing, and finally extruded through a polycarbonate filter with 400 nm pore size (Avestin, 11 passages). The experiment was started by diluting the proteoliposomes 20 times into a buffer containing 20 mM Hepes/Tris, pH 7.5, 200 mM NaCl, 3 μM valinomycin, and varying concentrations of KCl and Choline Cl were added in order to obtain the membrane potentials –78.06 mV, –39.03 mV and –26.02 mV, which are the calculated reversal potentials for hypothetical 4:1, 3:1 and 2:1 Na^+^:L-asp stoichiometries (35.0/26.4/19.2 mM CholineCl, 0/11.1/18.4 mM KCl).

After 1, 2 and 3 min the reaction was quenched with ice-cold quenching buffer containing 20 mM Hepes/Tris, pH 7.5, 250 mM Choline Cl and immediately filtered on nitrocellulose filter (Protran BA 85-Whatman filter). Finally, the filter was washed with 2 ml of quenching buffer. The flux of radiolabeled aspartate was measured by subtracting the amount of radiolabeled aspartate at 1 min from the amount of radiolabeled substrate at 3 min. The filters were dissolved in scintillation cocktail and the radioactivity was measured with a PerkinElmer Tri-Carb 2800RT liquid scintillation counter. The equilibrium, or reversal, potential, *E*_rev_, for each condition was calculated as described in [48].

## Results and Discussion

### Polarization Effects Explain Atypical Methionine Coordination of Na^+^

**Fig. 2:**
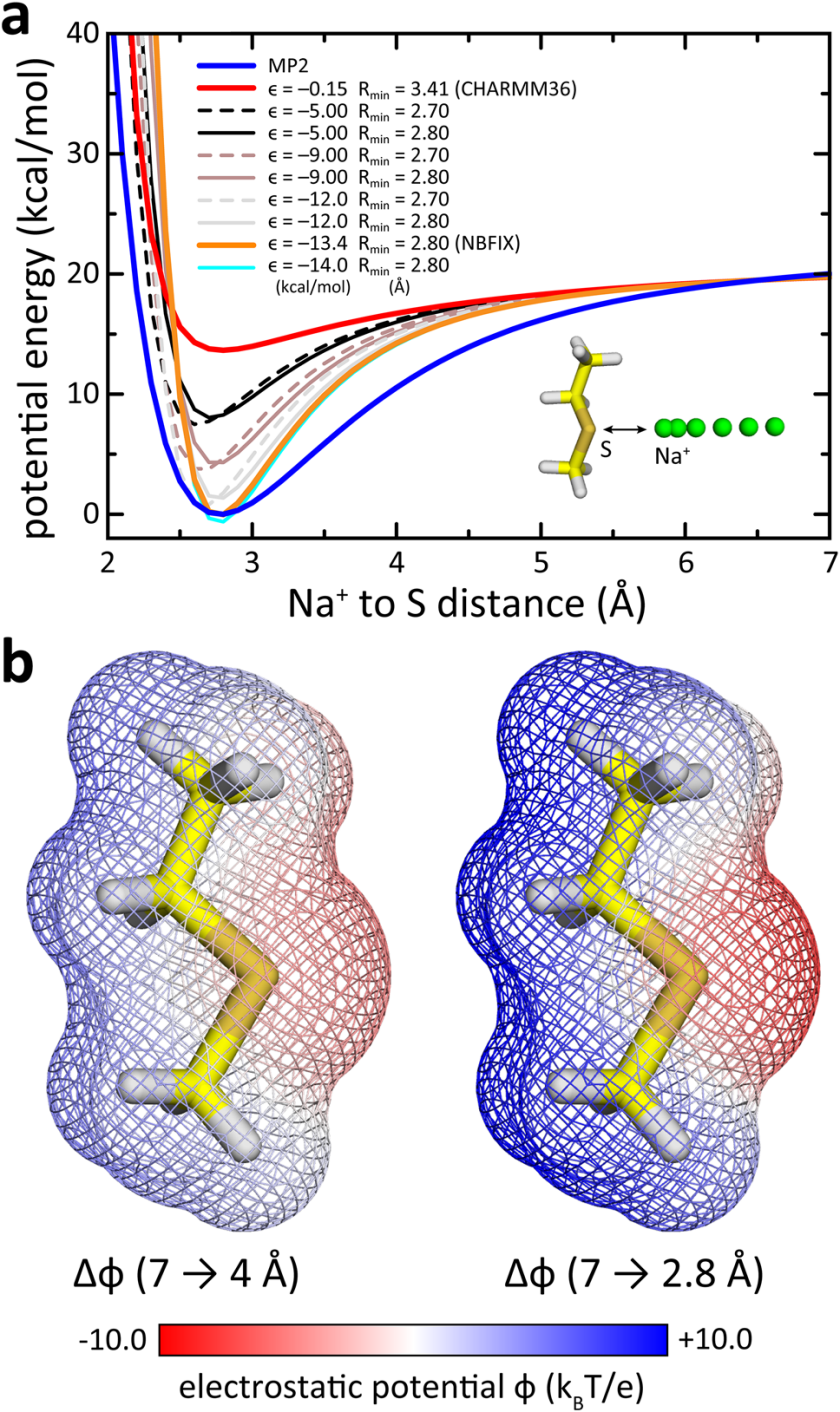
Polarization of the methionine side chain by close proximity with a sodium ion. **a** Change in the potential energy of a system comprising a methionine analog and a Na^+^ ion (*inset*) as the distance between the ion and the sulfur atom is varied from 7 to 2 Å. Quantum mechanical calculations at the MP2 level (*thick blue line*) are compared with values calculated with the standard CHARMM36 molecular-mechanics force field (*thick red line*), as well as with a series of modifications thereof in which the Lennard–Jones parameters used for the S-Na^+^ interaction are varied. Results are shown for values ranging from –5.0 to –14.0 kcal/mol and for *R*_min_ values from 2.7 Å (*dashed lines*) to 2.8 Å (*solid lines*). The values of the NBFIX correction implemented in this study are $\epsilon$ = –13.4 kcal/mol and *R*_min_ = 2.8 Å (*thick orange line*). **b** Change in the electrostatic potential that results from the change in the methionine analog atomic charges when the Na^+^ ion approaches the sulfur atom from 7 to 4 Å (*left*), or from 7 to 2.8 Å (*right*), i.e., the potential-energy minimum. The potential is mapped onto the surface of the analog (*mesh*). Larger contrast between positive (*blue*) and negative (*red*) values indicates a higher degree of polarization by Na^+^

As mentioned above, the observation that the methionine in the conserved NMDGT motif coordinates the Na^+^ ion in the Na2 site is, statistically speaking, highly atypical, given its hydrophobic character [49]. However, close-range contacts between methionines and the guanidinium and amino groups in arginines and lysines are common in known protein structures [50], as are interactions with carboxyl and carbonyl groups [51], and aromatic rings [51, 52]. This striking promiscuity indicates the sulfur atom in the methionine side chain is highly polarizable, i.e., it can alter its electrostatic character to better match its near environment. To examine whether this polarizability might also explain the interaction observed in the Na2 site in SLC1-family Na^+^-dependent transporters, we carried out a series of MP2-level quantum–mechanical calculations in which a Na^+^ ion was gradually brought closer to the sulfur atom in the methionine side chain analog methylthioethane, and evaluated the resulting interaction energy as well as the change in their electronic charge distribution. As shown in Fig. 2a (blue curve), this interaction is unequivocally attractive, favoring a contact distance between ion and sulfur of about 2.8 Å, which closely resembles what is observed in the structure of Glt_Tk_, namely 3.0 Å (on average). Further examination of these results with Natural Bond Orbital calculations reveals how the distribution of atomic charges in the analog is gradually altered as the Na^+^ ion becomes closer, with the sulfur becoming significantly more electronegative, and its flanking groups more electropositive (Fig. 2b).

**Fig. 3:**
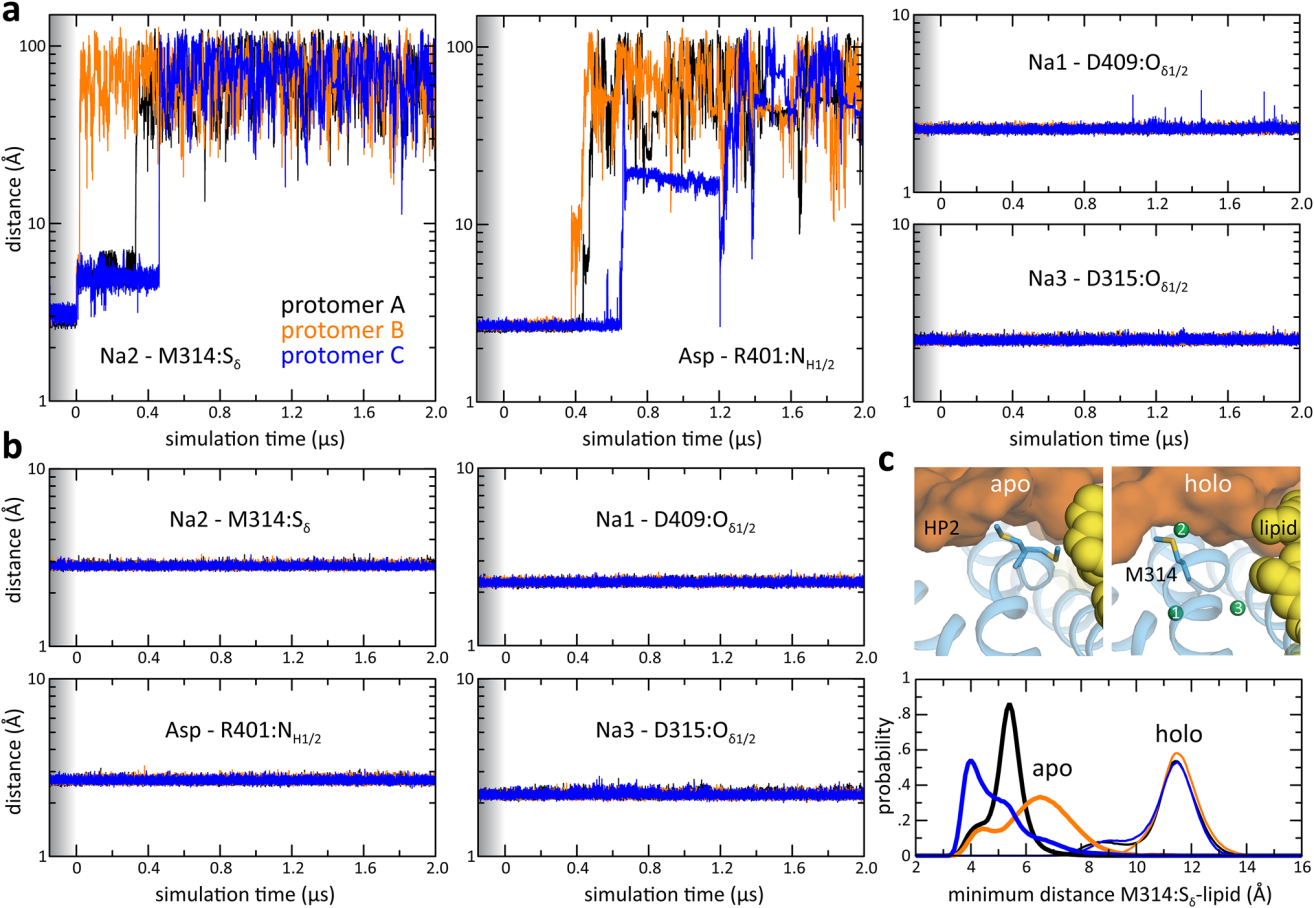
MD simulations of wild type holo-state Glt_Tk_ trimer with standard and corrected force fields. **a** Using standard CHARMM36, the Na^+^ ions at the Na2 sites rapidly unbind, followed by L-aspartate. The dissociation of the Na^+^ ion from Na2 (*left*) is measured by its distance to the sulfur atom in Met314, for each of the three protomers (*black, orange and blue lines*) and as a function of simulation time. The dissociation of L-aspartate was measured relative to the nearest terminal N atom of Arg401 (*center*). Ions at Na1 and Na3 are, in contrast, stably bound, as measured by the distance, respectively, to the Asp409 and Asp315 sidechain terminal O atoms (*right*). In all plots, the shaded area prior to *t* = 0 ns indicates the progressive reduction of conformational and distance restraints acting on the complex during the equilibration stages. **b** Using the NBFIX correction of Na^+^-methionine interactions, all Na^+^ ions and the substrate remain stably bound throughout the trajectory. **c** Orientation of the Met314 side chain toward the hydrophobic lipid bilayer is preserved in simulations of the apo transporter that also use the NBFIX correction. Upper: simulation snapshots are shown with the protein as cartoon helices, the sidechain of Met314 in sticks, HP2 as an orange surface and nearby lipid tails in yellow spheres. Lower: Distribution of distances between the sulfur atom and the nearest lipid tail atom in simulations of the apo (*thick lines*) and fully-occupied (holo, *thin lines*) transporter, with individual lines indicating different protomers in the same trajectory

To examine this interaction in the context of the structure of the transporter, we turned to all-atom molecular dynamics (MD) simulations. Specifically, we designed a simulation system including a wild-type Glt_Tk_ trimer in the outward-occluded conformation, with three Na^+^ ions and one L-Asp molecule bound in each protomer, embedded in a model phospholipid bilayer and a 100 mM NaCl solution; the resulting system thus comprises over 170,000 atoms (Fig. S1). To be able to calculate trajectories probing the microsecond timescale for such a system, and thus derive statistically significant data, we used a fixed-charged energy function, namely the CHARMM36 force field. A caveat of this and comparable force fields, however, is that they do not include electronic polarizability effects such as those discussed above. The CHARMM36 representation of methionine, for example, is adequate for a hydrophobic entity, but as shown in Fig. 2a (red curve), it fails to capture the emergence of an attractive interaction between Na^+^ and the sulfur atom, precisely because the atomic charges are constant. Accordingly, an MD trajectory of our holo-state Glt_Tk_ trimer using this force field showed irreversible dissociation of the Na^+^ ion at the Na2 site, in all three protomers, within 500 ns of simulation time (Fig. 3a). Further analysis shows this instability specifically results from a defective representation of the interaction with Met314, which breaks off immediately after the ion and side chain atoms are allowed to move freely (Fig. 3a). This observation is consistent with the conclusions of an independent simulation study of Glt_Tk,_ reported while this study was underway [53]. Indeed, irreversible dissociation of the Na2 ion appears to have been a common denominator of simulation studies of the mechanisms of both Glt_Ph_ and Glt_Tk_ [19, 54–56]. It is however straightforward to recognize this observation is artefactual. The dissociation constant of the Na2 site, while L-Asp is bound, has been estimated to be 10–100 µM [57], which is 3–4 orders of magnitude smaller than the concentration of free Na^+^ in simulation systems (~ 100 mM). The corresponding off-rate was also estimated at 100 per second [57]. It is clear, therefore, that irreversible dissociation of the ion in Na2 in microsecond scale simulations reflects a systematic methodological problem, rather than a mechanistically significant event.

A convenient approach to correct this type of force field deficiency, used previously for other problematic ion-protein interactions in CHARMM36 [58–61], is to customize the generic Lennard–Jones (LJ) potential by introducing parameters specifically designed to improve the interaction in question. As shown in Fig. 2a (orange curve) for the Na^+^-methionine interaction, optimized LJ parameters for S and Na^+^ indeed result in a very reasonable agreement between quantum–mechanical data and the now-corrected CHARMM36, particularly at close range. Reassuringly, a simulation of the holo-state Glt_Tk_ trimer carried out using this so-called NBFIX correction showed stable occupancy of the Na2 site (Fig. 3b), similar to the ions in the Na1 and Na3 binding sites (Fig. 3a, b). The presence of the ion at the Na2 site also stabilizes the L-aspartate in its binding site (Fig. 3b), unlike in the simulations without the force field correction, where the L-aspartate substrate dissociated subsequent to the Na2 ion (Fig. 3a). Stable binding of substrate and ions is arguably the expected outcome for microsecond-long simulations of this occluded-state wild-type transporter. It is important to note that this NBFIX correction does not impact any other interactions involving a methionine (or a Na^+^ ion), unlike what results from modification of the atomic charges [53]. Thus, all methionine residues in the simulation system, and Met314 in particular, preserve their primary character, which is hydrophobic. This factor is key because, in the apo-state of Glt_Tk_ and Glt_Ph_, Met314 reorients away from the Na2 site and becomes surrounded by other hydrophobic side chains [6, 37]. Owing to the specificity of the NBFIX correction, this intriguing duality is recapitulated in our simulations (Fig. 3c); indeed, the data shows that in apo Glt_Tk_, Met314 is also exposed to the hydrocarbon interior of the lipid bilayer.

### Met314 Does Not Define the Na^+^ Stoichiometry of Glt_Tk_

**Fig. 4:**
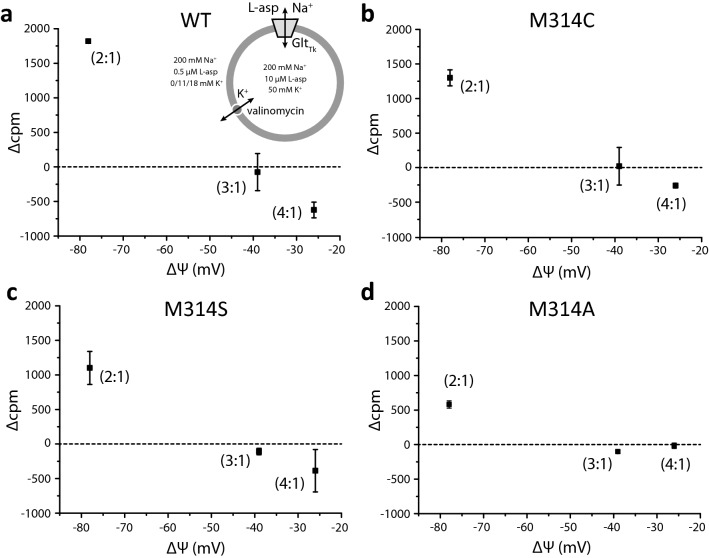
Determination of Na^+^:aspartate coupling stoichiometry using equilibrium potential measurements. Inset: Schematic of the experimental protocol used for proteoliposomes containing (**a**) wild type Glt_Tk_ protein and Met314 mutants (**b**) M314C, (**c**) M314S, and (**d**) M314A. The uptake or efflux of radiolabeled aspartate was determined by comparing the luminal radioactivity associated with the liposomes after 3 min of incubation with the radioactivity present after 1 min (Δcpm). For each membrane voltage probed, numbers in parentheses indicate the coupling stoichiometry that would be consistent with a zero-flux condition, if this condition had been observed. Error bars represent the ± SD obtained from 2 replicates

As mentioned, experimental mutation of the methionine in the NMDGT motif has clear effects on the function of both EAAT2/3 [21, 23, 33] and Glt_Ph_ [37–39]. Some of these observations, taken together with the nature of the Na^+^-methionine interaction discussed above, could be interpreted as evidence that this methionine is essential for defining the 3:1 stoichiometry of the coupled ion-substrate transport reaction. To examine this hypothesis experimentally, we determined the ion-substrate stoichiometry in wild-type Glt_Tk_ or in mutants in which Met314 was replaced by cysteine, serine, alanine or leucine, using purified protein reconstituted in proteoliposomes. To do so, we quantified the extent of Na^+^-coupled L-aspartate influx or efflux in conditions that would strongly favor efflux (a 20-fold outward gradient in L-Asp), were it not for an outward transmembrane electrical potential, experimentally generated as a potassium diffusion potential using the ionophore valinomycin (Fig. 4a inset). Because co-transport of Na^+^ and L-Asp moves positive charge across the membrane, this outward potential not only opposes L-Asp efflux, but can also drive uphill influx at sufficiently large (negative) values. The value at which no net flux occurs, referred to as the reversal potential, depends on the number of Na^+^ ions co-transported with L-Asp, and can be estimated a priori [48, 62, 63]. Here, we examined the effect of K^+^ diffusion potentials that would result in a zero-flux equilibrium in hypothetical reactions requiring 4, 3 or 2 Na^+^ ions in our specific experimental conditions. The results for WT Glt_Tk_ showed that this ‘reversal potential’ is –39 mV, which corresponds to a 3:1 stoichiometry (Fig. 4a). As expected, the smaller (less negative) potential allows for downhill efflux of L-Asp while the larger (more negative) potential has the opposite effect, and drives L-Asp into the liposomes, uphill. This result reproduces our previous measurements [15, 47] and is satisfyingly consistent with the structural data [19]. Surprisingly, however, analogous experiments for M314C and M314S demonstrate that the stoichiometry of the transport reaction is also 3:1 (Fig. 4b, c). M314A was only marginally stable in detergent solution and thus difficult to characterize conclusively, but the trends in the data suggest that its stoichiometry is also unchanged relative to WT (Fig. 4d). Unfortunately, M314L could not be characterized due to low expression levels.

**Fig. 5:**
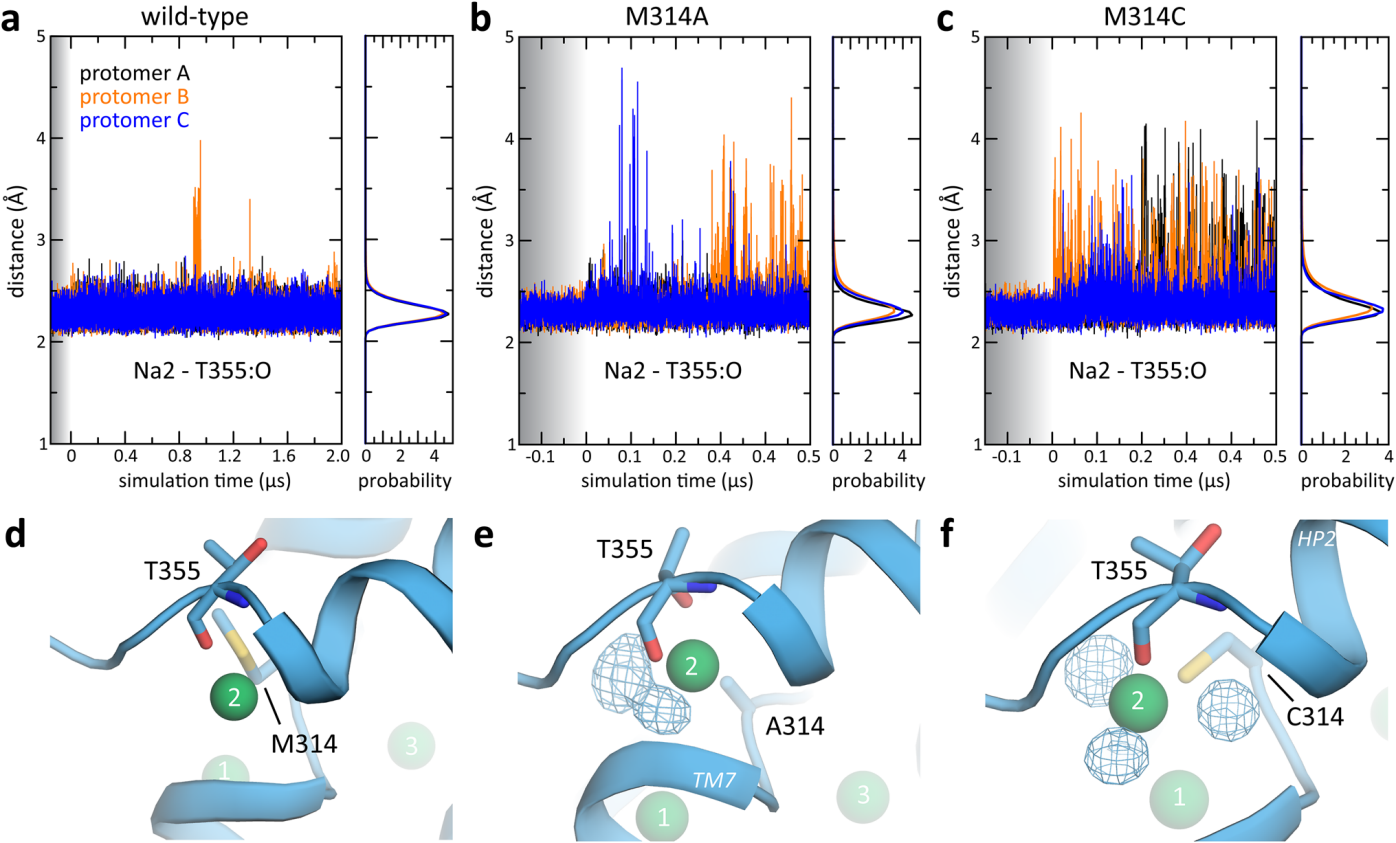
Ion and water occupancy in Glt_Tk_ mutants during MD simulations. **a**–**c** The coordination of the sodium ion in Na2 by the backbone carbonyl of Thr355 is monitored by the Na^+^-O distance, shown as a function of time (*left panel*) and as a distribution over time (*right panel*) for each of the three protomers in the simulation (*black, orange, blue*) for wild-type (**a**), M314A (**b**), and (**c**) M314C. **d**–**f** Close-up of Na2 binding sites in representative snapshots of the simulations of wild-type (**a**), M314A (**b**), and (**c**) M314C, indicating the location of the sodium ions (*green spheres*). Water molecules found to be involved in Na^+^ coordination in M314A and M314C are represented by density signals (*blue mesh*), directly calculated from the MD trajectories and averaged over the three protomers in the trimer. The protein backbone is shown as cartoon helices, and key side chains are shown as sticks

It is not self-evident how to rationalize these observations at the structural level. While serine side chains are often observed in Na^+^ binding sites coordinating the ion via their hydroxyl oxygen, cysteine and alanine side chains are uncommon [64]. Thus, to clarify these experimental results, we again turned to computer simulations. Specifically, for both M314A and M314C, we calculated MD trajectories analogous to those discussed above for the holo WT Glt_Tk_ trimer, i.e., with three Na^+^ ions and one L-Asp molecule initially bound to each protomer. (The S-Na^+^ interaction in the cysteine mutant was corrected following the same procedure as for methionine (Fig. S2a)). Consistent with the stoichiometry measurements, the Na^+^ ion at the Na2 site remained bound throughout these trajectories (Fig. 5b, c). In M314A, we observed that water coordinated the ion, along with the side-chain hydroxyl of Thr355, and thereby compensated for the lack of the thioether group in the wild-type structure, allowing the position of the ion to remain largely unchanged (Fig. 5a, b, d, e). In M314C, water molecules were also seen to participate in the coordination of the ion, as did the sulfur atom in the cysteine side chain (Fig. 5c, f), owing to polarizability effects comparable to those observed for methionine (Fig. S2b).

Taken together, measurements and simulations of WT and mutant Glt_Tk_ demonstrate that the Met314 sidechain is not required for occupation of Na2 by Na^+^, despite its role in ion coordination in the WT transporter. It seems reasonable to conclude, therefore, that the thermodynamics of the transport mechanism of Glt_Tk_, and by extension other Na^+^-coupled transporters in this family, is not fundamentally defined by the conserved methionine in the NMDGT motif through its contribution to the Na2 site. While this residue causes substrate and Na^+^ binding to be cooperative, this cooperativity is not essential for enforcing the stoichiometry of the transport cycle, implying that other factors more fundamentally control the coupling mechanism of this transporter.

### HP2 Occlusion as a Rationale for Stoichiometric Coupling

Previous studies of the Na^+^/Ca^2+^ exchanger NCX_Mj, also a secondary-active transporter, might provide clues into the nature of those factors. NCX_Mj translocates either 3 Na^+^ or 1 Ca^2+^ across the membrane [63], and thus functions as an antiporter. A systematic examination of the conformational energy landscape of NCX_Mj, using molecular simulations and X-ray crystallography, concluded that the interconversion between open and occluded states is the process that explains the stoichiometry of the transport cycle, i.e., the origin of the coupling mechanism [59]. Specifically, the authors found that occluded conformations, which are necessary intermediates in the alternating-access transition, are energetically accessible only when 3 Na^+^ or 1 Ca^2+^ are bound [59], partial or no ion occupancies, or H^+^ binding to the Na^+^/Ca^2+^ sites, do not permit occlusion, and accordingly do not catalyze transport. Consistent with this view, a recent study of Glt_Ph_ and a crystallizable EAAT1 variant suggests that the observation of K^+^ antiport correlates with the feasibility of HP2 closure after K^+^ binding [65].

**Fig. 6:**
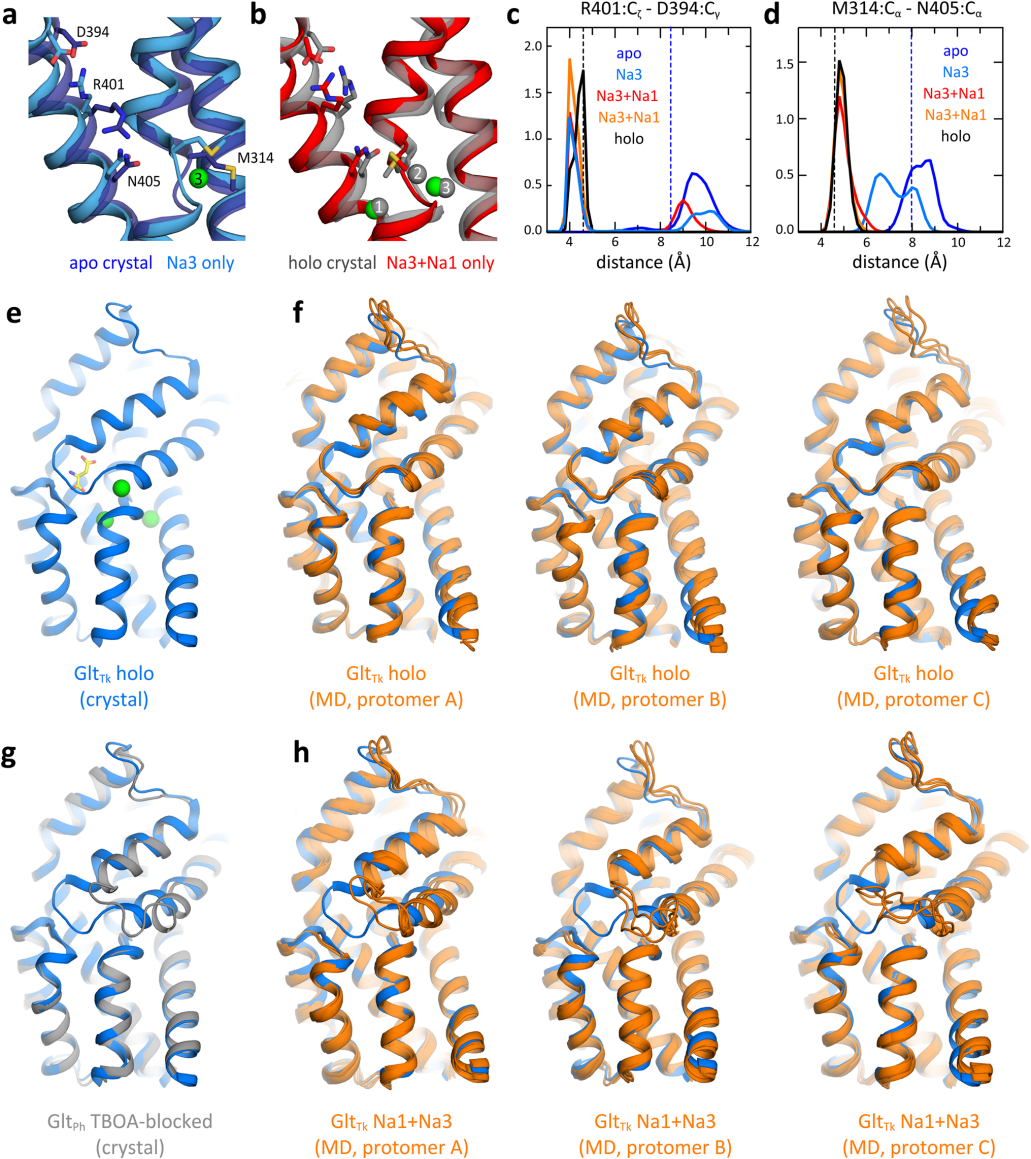
Conformation of Glt_Tk_ in a partially occupied state with Na^+^ ions at Na3 and Na1. **a** The crystal structure of the apo-state transporter (PDB entry 5E9S, *dark blue*) is superposed on a snapshot of the simulation of the apo state in which a single sodium ion (*green sphere*) was added to the Na3 site (*light blue*), with key side chains shown as sticks and labelled. **b** The crystal structure of the fully-occupied transporter (PDB entry 5DWY, *gray*) is superposed on a snapshot of the simulation in which two sodium ions were sequentially added at the Na3 and Na1 sites (*red*). **c** Distance between the side chain terminal carbon atoms of Arg401 and Asp394, shown as a probability histogram over time, for simulations of fully-occupied Glt_Tk_ (*black, holo*); the apo state (*blue*); a hypothetical intermediate occupied only at the Na3 site (*light blue*); and a state occupied at the Na3 and Na1 sites, either after release of Na2 and L-Asp (*orange*) or after addition of ions to the apo state (*red*). The formation of a salt-bridge between Arg401 and Asp394 primes the former to coordinate the substrate and requires binding of Na^+^ to Na3. The values of this distance in the crystal structures of apo (*blue*) and holo (*black*) transporter are shown as dashed vertical lines. **d** Distance between the Cα atoms of Met314 and Asn405 in TM8, shown as in (**c**). Binding of Na^+^ to Na1, with Na3 already occupied, recruits backbone atoms adjacent to Met314, and positions its sidechain for coordination of the third ion, at the Na2 site. **e** Crystal structure of the outward-facing holo-state of Glt_Tk_, highlighting the configuration of HP2 that occludes access to the Na^+^ and substrate binding sites. **f** HP2 remains closed during simulations of the holo-state transporter, as observed in the holo-state crystal structure (*blue*). Simulation snapshots (*orange*) are shown for each of the three protomers, taken at 1.0, 1.5 and 2.0 µs. **g** The crystal structure of the occluded holo-state of Glt_Tk_ (*blue*) is compared with the crystal structure of TBOA-bound Glt_Ph_ (*gray*) (PDB entry 2NWW). **h** HP2 opens during simulations in which only Na1 and Na3 are occupied, adopting a conformation comparable to that observed in the crystal structure of TBOA-bound Glt_Ph_. Simulation snapshots are shown (*orange*) for each of the three protomers, taken at 1.0, 1.5 and 2.0 µs, and superposed on the crystal structure of holo Glt_Tk_ (*blue*)

Our simulations of Glt_Tk_ provide support that this notion will apply to the substrate-Na^+^ stoichiometry in Glt_Tk_ as well. As noted above, in trajectories using conventional CHARMM36, we observe ready dissociation of Na^+^ from Na2 followed by L-aspartate (Fig. 3a). The order and timescale of these events is, as discussed, artefactual, but the resulting configuration, only occupied by Na^+^ in Na1 and Na3, is a mechanistically relevant state in physiological conditions [34–36]. In agreement with recently-published structures of inward-facing Glt_Tk_ [10] and outward-facing Glt_Ph_ [38], we observe that this outward-facing, partially-occupied state preserves many of the key features of the holo-state structure [19]. For example, the ion-pair between Arg401 and Asp394, which primes Arg401 for substrate coordination (Figs. 1b and 6a, c), is present. In addition, the configuration of Met314 in this state permits Na^+^ coordination at the Na2 site (Fig. 6b, d). Independent simulations initiated in the apo state, where Na^+^ ions were sequentially added at the Na3 and Na1 sites, corroborate these findings. These trajectories show that occupancy of Na3 alone controls the conformation of Arg401 (Fig. 6a, c), seemingly through their electrostatic repulsion. However, binding at Na3 alone is not sufficient to reorganize the backbone or side chain of Met314, even though Asn313 and Asp315, which flank Met314, both contribute side chains to the Na3 site. Only when Na1 is also occupied does Met314 reorient to match the geometry conducive to ion binding at the Na2 site (Fig. 6b, d).

This partially occupied state, however, lacks a central feature of the holo-state structure, namely HP2 in a configuration that occludes the binding sites (Fig. 6e). Invariably, the absence of L-Asp and the third ion at the Na2 site leads to the opening of this hairpin towards the extracellular space, which after 1 µs of simulation adopts a conformation closely resembling that observed in structures of Glt_Ph_ and Glt_Tk_ bound to blockers [5, 10] (Fig. 6g, h), and in a recently-reported structure of Glt_Ph_ in the same partially occupied state [38]. By contrast, extended simulations of the fully-occupied transporter (made possible by the NBFIX correction described above) demonstrate that the presence of all ions and the substrate correlates with HP2 favoring the occluded conformation (Fig. 6f). These results indicate that the energy landscape governing the dynamics of HP2, and by extension the occlusion of the transporter and the viability of the alternating-access transition, is indeed re-shaped by the occupancy or vacancy of the ion and substrate binding sites. In other words, opening of HP2 does appear to serve as the structural mechanism that prevents uncoupled transport, as suggested elsewhere [37, 38]. Further experimental and computational investigations will be required to continue to examine this hypothesis and fully clarify its molecular basis.

## Conclusions

Most secondary-active transporters couple the uptake or efflux of substrates to the co-translocation of ions in well-defined stoichiometries. This coupling is the defining functional feature of this class of proteins, as it permits them to harness transmembrane electrochemical gradients, usually of Na^+^ or H^+^, to power substrate transport, even against an opposing concentration gradient. The general principles that explain this coupling, however, remain to be clearly established. In Na^+^-dependent transporters of the SLC1 family, substrate and Na^+^ binding are strongly cooperative; therefore, it is intuitive to infer that this cooperativity in binding is what explains the substrate-ion coupling of the transport reaction. However, mutation of a conserved methionine in the so-called NMDGT motif greatly impairs this cooperativity in binding, and while it also slows down the turnover rate, our data shows that it does not impact the stoichiometry of the transport cycle. Therefore, we conclude that while cooperativity in binding can be a form of kinetic optimization, the stoichiometric coupling of substrate and ion transport is more fundamentally explained by other factors. A structural mechanism whereby only selected occupancy states permit the transporter to adopt occluded conformations, as it transitions between outward and inward-open states, is a likely explanation.

## Supplementary Information

Below is the link to the electronic supplementary material.

(DOCX 1776 kb)
