# Supplementary material for: On the Role of a Conserved Methionine in the Na+-Coupling Mechanism of a Neurotransmitter Transporter Homolog
Source: Neurochem Res. 2021 Feb 9;47(1):163–75. doi: 10.1007/s11064-021-03253-w (PMC8431971; doi:10.1007/s11064-021-03253-w)
Supplement: Supplementary file 1 — (DOCX 1776 kb) [file 11064_2021_3253_MOESM1_ESM.docx]

**Supporting Information**

**for**

**On the role of a conserved methionine in the Na^+^-coupling mechanism
of a neurotransmitter transporter homolog**

**Neurochemical Research**

Wenchang Zhou, Gianluca Trinco,
Dirk J. Slotboom, Lucy R. Forrest, José D. Faraldo-Gómez

Correspondence should be addressed to:

(DJS) [d.j.slotboom@rug.nl](mailto:d.j.slotboom@rug.nl), (LRF) [lucy.forrest@nih.gov](mailto:lucy.forrest@nih.gov)
and (JDFG) [jose.faraldo@nih.gov](mailto:jose.faraldo@nih.gov)

**Supplementary Tables**

**Table S1.** Structures of proteins hypothetically featuring S-Na^+^ interactions

| PDB identifier | Function | Ion residue number | Distance(Å) | Protein residue number* |
| --- | --- | --- | --- | --- |
| 3QJX | Aminopeptidase | 892 | 3.1 | Met260 |
| 5BPH | D-alanine-D-alanine ligase | 402 | 3.2 | Met95 |
| 5OGX | P450 aryl-O-demethylase | 403 | 2.8 | Met31 |
| 3FGW | Unknown | 610 | 3.0 | Cys249 |
| 3MOS | Transketolase | 628 | 3.2 | Cys468 |
| 3ROX | D-serine deaminase | 454 | 3.2 | Cys309 |

*Where multiple chains were present, the interactions listed are in the chain with identifier A.

**Supplementary Figures**

**
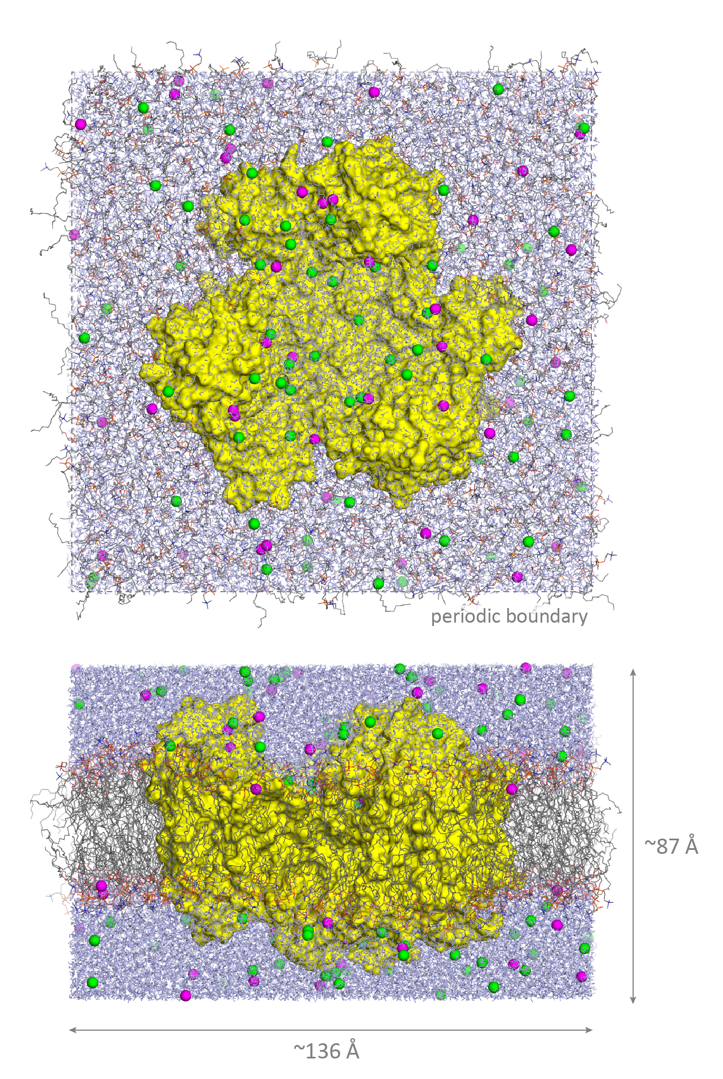
**

**Figure S1.** Molecular dynamics simulation of outward-facing Glt_Tk_. The figure shows the final snapshot of the trajectory calculated for the hole state, viewed from the extracellular side (*upper panel*) and along the membrane plane (*lower panel*). The transporter (*yellow surface*) is immersed in a POPC lipid bilayer (*lines*) and a solution containing 100 mM NaCl plus counterions neutralizing the total system charge (*green/magenta/purple*). The total number of atoms is approximately 170,000. All boundaries are periodic.


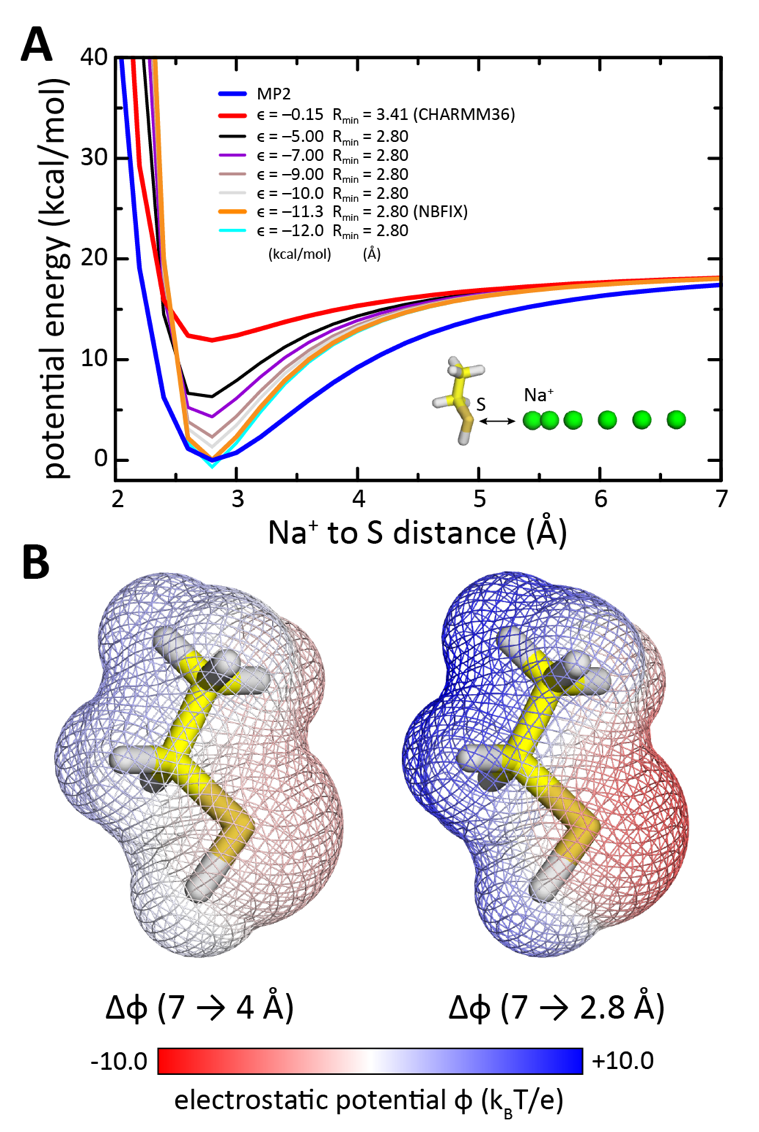

**Figure S2.** Polarization of the cysteine side chain by close proximity with a sodium ion. **(A)** Change in the potential energy of a system consisting of a cysteine analog and a Na^+^ ion (*inset*) as the distance between the ion and the sulfur atom is varied from 7 to 2 Å. Quantum mechanical calculations at the MP2 level (*thick blue line*) are compared with values calculated with the standard CHARMM36 molecular-mechanics force field (*thick red line*), as well with a series of modifications thereof in which the Lennard-Jones parameters used for the S-Na^+^ interaction are varied. Results are shown for 𝜖 values ranging from –5.0 to –12.0 kcal/mol, with *R*_min_ = 2.8 Å (*thin* *solid lines*). The values of the NBFIX correction implemented in this study are 𝜖 = –11.3 kcal/mol and *R*_min_ = 2.8 Å (*thick orange line*). **(B)** Change in the electrostatic potential that results from the change in the cysteine analog atomic charges when the Na^+^ ion approaches the sulfur atom from 7 to 4 Å (*left*), or from 7 to 2.8 Å (*right*), i.e. the potential-energy minimum. The potential is mapped onto the surface of the analog (*mesh*). Larger contrast between positive (*blue*) and negative (*red*) values indicates a higher degree of polarization by Na^+^.

**
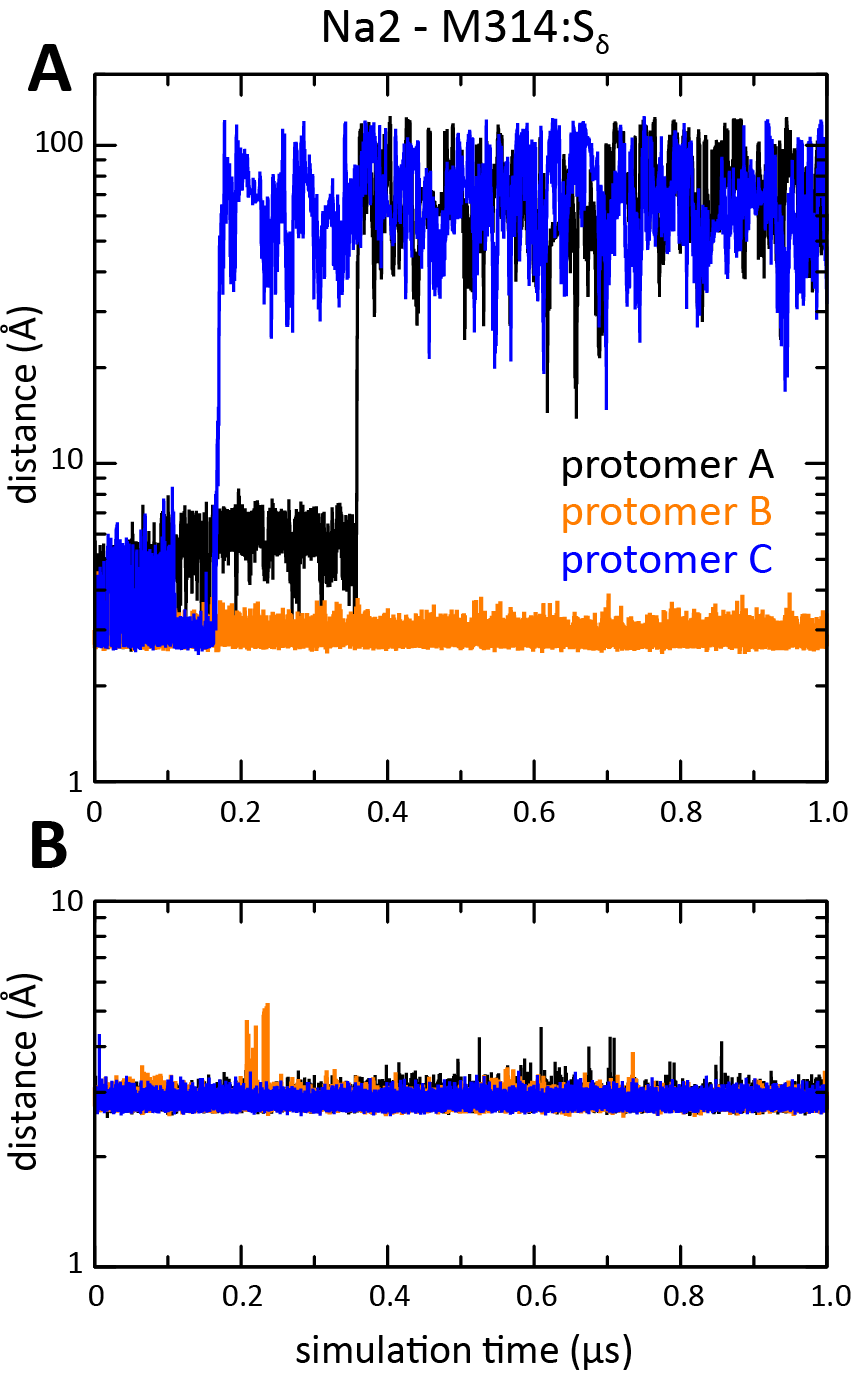
**

**Figure S3.** MD simulations of wild-type holo-state Glt_Tk_ trimer carried out with alternative corrections of the Lennard-Jones interaction between Na^+^ and the methionine S atom. As in Fig. 3, the plots represent the stability of the ion that was experimentally observed at the Na2 site in terms of the time-series of the distance between the ion and the S atom of Met314, during a 1 µs-long MD trajectory. (**A**) Data obtained for ε = –5.0 kcal/mol and R_min_ = 2.80 Å (black solid curve in Fig. 2A); this correction is insufficient to capture the polarization effects that stabilize the Na^+^-methionine contact, and accordingly, fails to reproduce the expected occupancy of the Na2 site, similarly to the uncorrected CHARMM36 forcefield. (**B**) Data obtained for ε = –9.0 kcal/mol and R_min_ = 2.80 Å (brown solid curve in Fig. 2A). Although this correction underestimates the gas-phase interaction between Na^+^ and methionine, as computed with MP2-level QM calculations (blue curve in Fig. 2A), it is a plausible alternative to the full correction because gas-phase polarization effects will be somewhat diminished in the context of a protein binding site, wherein the ion is coordinated by multiple ligands, with competing effects. Indeed, the simulation data obtained with this correction demonstrates that the Na2 site remains occupied throughout the trajectory, in all three Glt_Tk_ protomers, as observed for the full correction.
